# Refugee Mothers Mental Health and Social Support Needs: A Systematic Review of Interventions for Refugee Mothers

**DOI:** 10.5964/ejop.4665

**Published:** 2022-08-31

**Authors:** Kim Roger Abi Zeid Daou

**Affiliations:** 1 McGill University, Montréal, Québec, Canada; University of Neuchâtel, Neuchâtel, Switzerland

**Keywords:** refugee women, at-risk populations, mental health, humanitarian crisis, motherhood, community interventions

## Abstract

Refugee mothers endure and are at risk for depression, post-traumatic stress, suicidality, and anxiety. There is a gap in the literature regarding interventions for refugee mothers’ mental health and well-being. Interventions involving refugee mothers rarely provide adequate support for refugee mothers’ specific mental health needs and challenges. This paper presents empirical evidence to contextualize the risks refugee mothers face, such as gender-based violence, mental health challenges, and language barriers. Then, the paper provides a critical systematic review of interventions conducted with refugee mothers. The critical systematic review suggests that creating and providing a safe space, being a linguistic liaison, community-building, and advocating for refugee mothers’ needs are emergent protective factors for refugee mothers. Finally, based on the review, recommendations for future interventions followed.

Although there is evidence that refugee mothers experience mental health issues, there is a gap in the literature on interventions supporting mental health and adjustment for refugee mothers. Indeed, refugee mothers experience depression, anxiety, post-traumatic stress, and suicidality (Rahman & Hafeez, 2003; Schweitzer et al., 2018). Intervention programs and services for refugee populations seldom provide adequate support to the mental health needs and challenges of refugee mothers (Dybdahl, 2001; Jesuthasan et al., 2019; Milkie et al., 2020). However, the United Nations High Commissioner for Refugees (UNHCR) identifies the urgent need for specialized support for refugee mothers (United Nations High Commissioner for Refugees, 2014a). Thus, this paper will begin by presenting a review of studies that have explored the mental health issues, experiences, or risks refugee mothers face. This will be followed by a critical systematic review of interventions conducted with refugee mothers to determine the match and/or discrepancy between needs and interventions. Finally, based on this review, recommendations for future interventions will follow.

## Vulnerabilities of Refugee Women and Mothers

### Gender-Based Violence

Refugees are exposed to difficult and traumatizing experiences, which affect their psychological and emotional well-being, making them highly vulnerable to mental health problems and emotional distress (Agic et al., 2016; Crowley, 2009). Moreover, the UNHCR states that refugee women and girls are extremely vulnerable (United Nations High Commissioner for Refugees et al., 2016). The experiences of refugee women and girls throughout the resettlement process are different from men’s experiences since many of them are subjected to gender-based violence and persecution (Pittaway & Bartolomei, 2001; Pulvirenti & Mason, 2011; United Nations High Commissioner for Refugees et al., 2016). Women and girls who are normally protected because of their gender now lack that protection and are at risk for gender-specific human rights violations (United Nations High Commissioner for Refugees, 2014b; United Nations High Commissioner for Refugees et al., 2016). Refugee women and girls are especially vulnerable to sexual gender-based violence in refugee camps, where they are often required to walk long distances to get important resources such as firewood. Moreover, they are still at dramatically elevated risk for gender-based violence through migration routes in Europe and even after resettlement (Robbers et al., 2016). The lifetime prevalence of sexual violence against girls and women over 15 in the general population was 11%, while it reached 69.3% of migrants and refugees (Keygnaert et al., 2012). Additionally, gender-based and sexual violence has long-term impacts on refugee women and girls’ mental health and well-being including post-traumatic stress disorder (PTSD), depression, anxiety, and suicidality (Robbers et al., 2016).

Gender-specific human rights violations have harmful impacts on refugee women’s physical and psychological states, which are further exacerbated by the challenges of resettlement (Deacon & Sullivan, 2009; Hynes & Cardozo, 2000; Miller et al., 2002).

### Mental Health Challenges

Refugee women have been found to have especially high rates of psychiatric symptoms. Indeed, the traumatic experiences and post-migration challenges refugee women endure have the potential to reinforce increased vulnerability to psychiatric symptoms and distress including symptoms of trauma, anxiety, depression, somatization, and PTSD (Schweitzer et al., 2018). Given the higher risk of experiencing mental health issues as a refugee during childbearing age (Korukcu et al., 2018), refugee mothers are especially vulnerable to developing mental health issues due to their rapidly changing living situation and circumstances (Degni et al., 2014). For instance, refugee mothers in camps face a high risk of developing a mental disorder, such as depression, and to experience suicidal ideation. Indeed, a study revealed that 36% of the mothers in camps, were diagnosed with a mental disorder, 91% percent of which reported having had suicidal thoughts in the past month **(**Rahman & Hafeez, 2003).

Furthermore, Schweitzer and colleagues (2018) found that the responsibilities of caring for a child as a refugee represent a risk factor for refugee mothers experiencing higher levels of trauma symptoms and contribute to the development of psychiatric problems such as anxiety and somatic symptoms (Schweitzer et al., 2018). Newly resettled mothers are less likely to seek out professional services due to various barriers including stigma, language, poor knowledge of community services, and prioritizing their children’s needs to the detriment of their own well-being (Nicholson et al., 1998); refugee mothers’ focus on supporting their children and coping with these barriers leave them at an elevated risk for mental health problems (Betancourt et al., 2015). Thus, refugee mothers face a unique set of challenges that require specialized evidence-based interventions.

### Language Barriers

Moreover, the language barrier through the resettlement process is an impediment to refugee women’s access to services which in turn can negatively affect their psychological well-being (Hou & Beiser, 2006). This barrier is especially salient in the post-resettlement context where refugee women seldom have enough time and resources to address their educational needs and learn the language of the host country (Watkins et al., 2012).

Furthermore, the language barrier disproportionately affects women, as immigrant men have been shown to achieve better proficiency of the host country’s language than women due to inequalities in social and educational opportunities (Hou & Beiser, 2006). A Canadian study found that refugee women’s English fluency was longitudinally associated with higher rates of employment and lower rates of depression (Beiser, 2009). In a study conducted in Germany, difficulties related to the language barrier were found to be a widespread concern for Syrian refugees which impeded their integration, their ability to socialize, and their access to proper healthcare (Green, 2017). Similarly, an Australian study found that having limited English proficiency increased refugee mothers’ risk for marginalization, isolation, and family dysfunction, while a stronger proficiency made them more comfortable accessing mainstream services (Riggs et al., 2012). Therefore, it is critical that language education services consider the needs of refugee mothers to support their host-country language fluency.

The mental health difficulties of refugee mothers such as depression, anxiety, trauma, suicidality (Rahman & Hafeez, 2003; Schweitzer et al., 2018), require specialized services because more general mental health services do not simultaneously address their cultural and linguistic backgrounds, their need for community, their mistrust and difficulties navigating healthcare systems in host countries, and the unique adverse experiences brought about by being a mother through the process of forced displacement (McLeish, 2005; Schweitzer et al., 2018; Tsai et al., 2017). Thus, the present paper will consist of a systematic review of interventions for refugee mothers while using specific inclusion and exclusion criteria, identify gaps in the literature, and provide research contributions and practical implications and recommendations for future evidence-based interventions and research.

## Method

### Search Strategy

**Figure 1:**
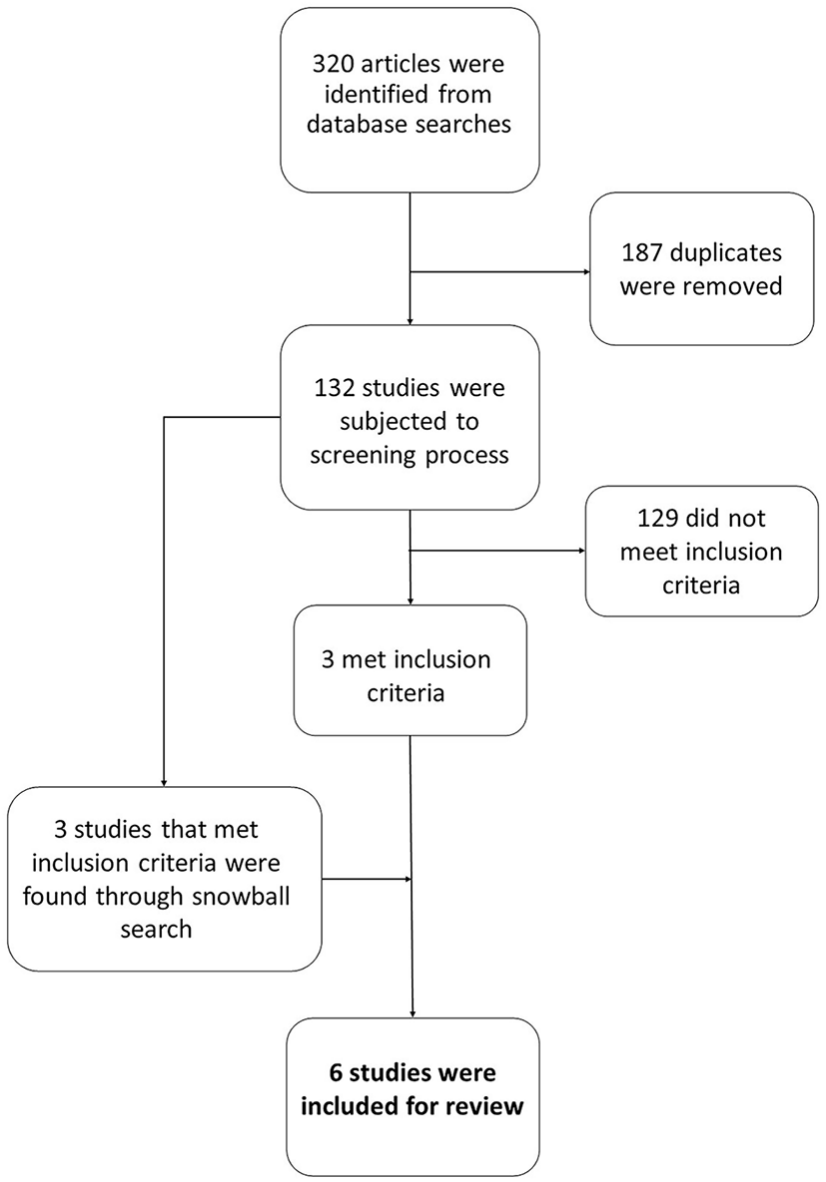
Search Strategy Flowchart

An electronic search was performed on PsycINFO (the year of 1806 to March 16, 2020), MEDLINE (the year of 1946 to March 16, 2020), and Web of Science Core Collection (accessed March 16, 2020). The search was limited to articles in the English language. The time periods were selected to encompass the year the database was founded to the present date. The following search was performed on all three databases: refugee* AND mother* AND interven*. The asterisk was used in order to truncate search words and include articles that use similar words such as *mothers* or *motherhood, refugees,* and *intervention* or *intervening.* A second researcher simultaneously conducted the search. The screening process, choice of articles, inclusion and exclusion criteria involved both researchers to ensure inter-rater reliability. Studies were included based on the following inclusion and exclusion criteria: (a) presents an original intervention, (b) focuses on refugee populations, (c) includes or targets mothers, (d) differentiates between data gathered with mother and father refugees, and (e) is not solely medical in scope, (see Figure 1).

## Results

The study selection process comprised three databases and incorporated specific inclusion and exclusion criteria. This search resulted in 132 studies. 112 studies were excluded because they did not present an original intervention, with many being qualitative in-depth interviews, research on refugee mothers’ reproductive health, vaccine research, and post-partum depression data research. Two studies were excluded because they did not focus on refugee populations, nine were excluded because they did not target or include refugee mothers, five were excluded because they did not differentiate between the needs of refugee mothers and fathers, and one study was excluded because its scope was solely medical. Three studies were included from this search, and three additional studies were found through a subsequent snowball search, which entails consulting the bibliography for relevant papers. Six relevant studies that involved a total of approximately 350 participants were included in the review.

### Interventions for Refugee Mothers

#### Children and Mothers in War: An Outcome Study of a Psychosocial Intervention Program

The aim of the psychosocial mother-child intervention was mainly to improve child development and well-being through better mother and child interactions, support and education (Dybdahl, 2001). The participants were 87 Bosnian-displaced mother-child dyads. They were divided into an intervention group (psychological intervention and medical care) and a non-intervention group (medical care only). The intervention was based on therapeutic discussion groups for traumatized women in the context of war and the International Child Development Program. The group discussions also involved topics related to mothers’ mental health such as symptoms of PTSD, traumatic experiences, exposure to violence, and other adverse experiences lived in refugee camps.

Before the start of the intervention, both the mothers and children’s psychological, cognitive and physical health were monitored. The study was culturally specific, and Bosnian mental health specialists reassessed and reviewed the instruments used in the study and intervention for cultural specificity and appropriateness.

Mothers’ trauma and wellbeing were measured using the Impact of Event Scale (IES) and the mothers’ interviews pre-intervention and post-intervention provided demographics, perceived social support, and living conditions information. Mothers who were in the intervention group perceived more social support after the intervention and felt they had people to go to for support and advice. Indeed, there was a significant increase in perceived social support between pre-test and post-test in the intervention group, while there was a slight decrease in the non-intervention group (Dybdahl, 2001).

There was a non-significant increase in total social support scores; pretest (*M* = 4.4) smaller than post-test; (*M* = 5.0), *p* > .05. There was a significant increase in social support for “advice” scores; pretest (*M* = 4.7) smaller than post-test; (*M* = 5.4), *p* < .05. There was a non-significant increase in mother’s well-being for “today”, pretest (*M* = 4.1) smaller than post-test, (*M* = 4.4), *p* > .05, and there was a significant increase in mother’s well-being for “usually”, pretest (*M* = 3.5) smaller than post-test, (*M* = 4.6), *p* < .05. There was a non-significant decrease in mother’s well-being for “prefer”, pretest (*M* = 6.2) bigger than post-test, (*M* = 5.8), *p* > .05. There was a significant decrease, pretest (*M* = 71.2) bigger than post-test, (*M* = 56.1), *p* < .05 in total IES scores and on the hyperarousal symptoms, pretest (*M* = 22.7) bigger than post-test, (*M* = 16.7), *p* < .05 subscale for mothers in the intervention group, which was significantly larger than the decrease observed in the control group. However, there was no significant difference between the decrease observed in the intervention group and the non-intervention group on the avoidance and intrusion symptoms subscales.

Dybdahl (2001)’s study is a good example of an intervention program that addressed the needs and prognosis of refugee mothers. However, the data gathered focuses heavily on the children and provides limited insight into the impact of the intervention on mothers. While mothers’ mental health problems are taken into consideration in the conception of the intervention, it is unclear whether it is efficient in promoting their well-being. Mothers’ wellbeing was only lightly discussed in relation to children’s wellbeing (Dybdahl, 2001). It is important to note that though the study demonstrated a decrease in mothers’ IES scores, those outcomes were not discussed sufficiently. Indeed, the researcher acknowledged that discussing mothers’ mental health in detail goes beyond the scope of the paper, but that the IES measures revealed a high level of distress both before and following the intervention, and that the IES scores were related to trauma exposure. It is worthwhile to highlight that the study aimed to be culturally specific and consulted numerous Bosnian mental health workers as it has been shown that culturally specific approaches are beneficial for refugee populations (Tsai et al., 2017; Williams & Thompson, 2011)

#### Collaborative Health Education for Somali Bantu Refugee Women in Kansas City

The goal of this study was improving the health literacy of Somali Bantu refugee mothers. Eleven Somali Bantu refugee mothers resettled in Kansas City were recruited through a resettlement agency to participate in a health education program held weekly over 12 months consisting of a total of 42 sessions of 90-minutes each (Mulcahy et al., 2019). Rooted in community-based collaborative action research (CBCAR), the small-group sessions took into account the needs and interests of mothers, and consequently included various topics such as family health, nutrition, sexuality, prenatal health, child safety, and mental health. To gain an understanding of the health narratives of refugee mothers through the resettlement process, each mother also participated in an individual interview. Additionally, they completed questionnaires pertaining to their health behaviors, the content of the testing sessions and whether they recalled the information presented.

Nutrition was the most requested and discussed topic. Pearson co-efficient analyses revealed a positive correlation between the number of times a topic was presented and the number of women who retained the topic (*r*  = .852, *p* < .01). Nutrition was the topic with the highest retention, as it was the topic that was presented the highest number of times. The women were interested in discussing nutrition in relation to their own health and that of their child, and nutrition in relation to prenatal health. PTSD symptoms were discussed in terms of the women’s exposure to potentially traumatizing experiences, hardship, and violence, but were not endorsed by participants during individual interviews. Finally, the women expressed being generally satisfied with their healthcare access, interactions with doctors, and access to interpreters.

Mulcahy and colleagues (2019) conducted the study to improve the health literacy of Somali Bantu refugee mothers. However, the mothers were asked about PTSD symptoms as “feeling sad” by culturally representative professionals with experience working with refugees. Moreover, recommendations in the literature highlight the importance of providing refugees with opportunities to cope with distress and negative affect without forcing them to relive their traumatic experiences (Hansen & Houston, 2016). In addition, women did not endorse PTSD symptoms when individually interviewed, and the results of the study do not provide information regarding their mental health through the course of the intervention. No other symptoms of mental illness or psychological distress were discussed.

Furthermore, despite holding a full session on the topic of mental health, the impact of the session on participants was not discussed in the paper. Despite claiming that some sessions focused on mental health, mental health was not discussed in the article beyond symptoms of PTSD. Additionally, it is stated that retention of the material on mental health was *null*, and that they found an “inconsistency between personal narrative and recognition of symptoms” (Mulcahy et al., 2019, p. 5). Unfortunately, mental health remains one of the biggest outcomes of concern for refugee mothers and this study did not properly assess nor intervene on refugee mothers’ mental health symptoms.

#### A Home-Based Intervention for Immigrant and Refugee Trauma Survivors

Visiting Moms is a program for high-risk refugee and immigrant mothers and their infants based at the Massachusetts General Hospital Chelsea Health Care Centre (Paris & Bronson, 2006). The intervention program focused on the needs of both mothers and infants and their functioning as a dyad and adopted a community-based approach in which paraprofessionals (obstetricians, midwives, pediatricians, and psychotherapists) provided at-home visits to participating new mothers. To be eligible, due to limited resources, immigrant and refugee mothers underwent a screening process to establish existing risk factors including severe depression, isolation, trauma, risk of child abuse, health needs, and safety of the family environment. 105 mothers participated in the study. The intervention comprised multiple aspects such as education on child development, family advocacy, and social support.

In its conception, the intervention program was claimed to be highly individualized, provided holistic support for mother-child dyads, and was rooted in evidence-based practice as well as local realities. However, no details were provided in the article to supplement those claims.

Parent-child interactions, and additional stressors, such as poverty and language barriers, were assessed and taken into consideration. The home visitors intervened by using relationship-based model (Lieberman, 2004; Paris & Bronson, 2006). The first step of the intervention was for the home visitor to listen to the mother, which made home visitors realize that the families needed basic supplies. The paper does not specify how information was elicited from mothers. The second step was developing a working alliance. The alliance was developed as the mother and the home visitor got to know each other every visit. The home visitor engaged in modeling and self-disclosure as communicational tools to develop an alliance, to teach the mother certain practices, and to make the mothers more comfortable opening up to the home visitor about their troubles. The last step was expanding the relationship to community supports, such as giving the mothers access to English classes or helping them get a library card. No data was collected to assess outcome measures, as the paper consists of a description of the program.

One positive facet of the intervention was advocacy: the home visitors advocated for the mothers and connected them to resources and to the community. The home visitors developed a working alliance with the mothers by supporting their autonomy and fostering their competence. Autonomy was fostered by the home visitor teaching the mother skills that could be useful in the host country. This is important to highlight, as the literature review previously mentioned that newly resettled refugee mothers are less likely to seek professional services because of various challenges, such as stigma and poor knowledge of community services (Nicholson et al., 1998).

Thus, the Visiting Moms intervention attempted to tackle important issues refugee mothers face, such as loneliness and lack of knowledge and access to resources. However, the outcomes of Visiting Moms were not formally studied. Therefore, there is no empirical evidence of the efficacy of the program and its impact on participating refugee mothers. Finally, the paper implied different cultural practices may be dangerous for the children, and that they taught mothers to practice those safely. However, the paper did not present an empirical method to define what is safe and unsafe for the children and operated under the assumption that western child-rearing practices are superior to other cultures.

#### The Moving Forward Project: Working With Refugee Children, Youth, and Their Families

The Moving Forward Project was an intervention program for refugee families based in Saskatchewan, Canada (White et al., 2009). The objective of the intervention was to provide knowledge and skills to refugee parents and youth to support them in efficiently addressing issues pertaining to trauma in the context of resettlement. The second goal was to bring awareness and knowledge of the resources and programs available to refugees and immigrants. The third goal was to improve service providers’ capacities, so they can respond better to refugee families’ needs. The goals were to be achieved through education, group discussions, resource development and dissemination. The first intake group included seven to ten families from Sudan and Afghanistan, and the second in-take session included eighteen families from Colombia, Afghanistan, Sudan, Burma, Rwanda, Congo, Egypt, Mongolia, Bosnia, and Burundi. There were six-week sessions held with the participants.

The intervention’s group sessions focused on topics such as the impact of trauma on the family, positive coping skills, and problem-solving skills. It should be noted that the authors did not disclose their data collection process or method. Nevertheless, the study highlighted the importance of relationship-building and support between women. Indeed, they stated that according to theories on group principles, participants would learn they are not alone in their experiences, and they could consequently learn from each other and support each other. They found that the language barriers limited the efficacy of the intervention and that the mothers preferred to talk about sociocultural integration and making Canadian friends instead of talking about past experiences.

Finally, they found that the session ended up being *gatherings*, a construction of a safe space for sharing, and that making the groups open to newcomers contributed to the construction of a safe space where mothers cried, talked, bonded, and laughed together. A safe space in this context refers to any physical space where refugees can feel physically and emotionally safe to express themselves and build social networks (United Nations High Commissioner for Refugees, 2014b).

A problematic aspect of the study was how the researchers stated that the language barrier and the refugees’ tendency to speak to each other in their mother tongue was a difficulty during the sessions. As the literature has shown, refugees have language difficulties when they arrive to their host country (Green, 2017; Watkins et al., 2012). Conducting an intervention with refugees in the host country language, in a language they either do not know or are struggling with, is a methodological problem. The authors sought to teach the refugee mothers skills (e.g., problem-solving and coping skills to deal with trauma) but claim the refugee mothers discussed their future in Canada instead. However, it is possible that the inability to communicate comfortably in their mother tongue contributed to a general discomfort resulting in hesitancy to share more personal information, although this would need to be evaluated. Certainly, evidence stresses the importance of using a professional interpreter with refugees when professionals do not speak the language, as the language barrier is a major challenge in providing accurate and adequate healthcare (Kavukcu & Altıntaş, 2019; Williams & Thompson, 2011). Finally, though the intervention claimed to delve into trauma, the researchers failed to consider the more specific mental health challenges of refugee mothers and their willingness and psychological readiness to discuss those issues in a language they are not comfortable in.

#### Sweet Mother: Evaluation of a Pilot Mental Health Service for Asylum-Seeking Mothers and Babies

O’Shaughnessy and colleagues (2012) evaluated a novel pilot intervention for refugee mothers and their infants called Sweet Mother. The objective of Sweet Mother was to promote participants’ mental health who have been exposed to adverse circumstances through the resettlement process during the perinatal period. A total of 13 mother-child dyads participated in the study. However, only seven dyads attended many or all of the 21 group sessions, while six dyads only attended between one and four sessions. The intervention was rooted in attachment theory and focuses on building on the mothers’ strengths to foster the development of a positive mother-child relationship. Moreover, it adopted a community-building approach to mitigate the negative impact of being separated from their home communities and adopted a participatory approach where children’s needs helped shape the group sessions.

The intervention consisted of therapeutic infant-mother group sessions lead by specialists. Mothers also participated in individual interviews, in reflective group discussions, and completed questionnaires pertaining to their relationship with their babies at each session. Thematic analysis of the reflective exercises revealed the mothers had an overall positive experience with the intervention. They expressed appreciating a new sense of *togetherness*, highlighted the importance of their babies socializing with other babies, expressed feeling safe in this group, learned about motherhood and parenting, and valued discussing and strengthening their relationship with their babies. Finally, the CARE-Index, an observation measure for adult-child dyads, revealed that two participants improved the quality of their mother-infant interactions from “seriously compromised” to “of concern”, while two mothers remained at the cusp of “of concern” and one mother’s scores increased within the “good enough” range (O’Shaughnessy et al., 2012).

It is important to note that the results of the Sweet Mother intervention provided an encouraging insight into an intervention model for at-risk refugee mothers with young children. However, as this was pilot study, the scope of the results was quite limited. Only five mothers were evaluated using the CARE-Index, limiting our understanding of the impact of the intervention on mother-child dyads. Additionally, one of the main objectives of the study was to “support maternal mental health by reducing isolation and increasing access to community resources” (O’Shaughnessy et al., 2012, p. 217). Yet, no screening for exposure to traumatic experiences or psychiatric symptoms was performed. Likewise, no identified outcomes were examined vis-a-vis the mothers’ psychological well-being. The methodology of the study lacked a direct measure of participating mothers’ mental health and general well-being and thus did not properly align with its outlined objectives. Nonetheless, the results of this pilot study and the positive response from participants provide important information on the needs of refugee mothers and the feasibility of such interventions.

#### I Think Someone Is Walking With Me: The Use of Mobile Phone for Social Capital Development Among Women in Four Refugee Communities

The goal of the intervention was the development of social capital by providing refugee mothers and women a phone. The main component of the intervention was examining the effects of acquiring and utilizing social capita. The intervention encompassed face-to-face peer support training sessions and mobile phones for the timespan of one year. 111 Afghan, Burmese, and Sudanese refugee women and mothers residing in Melbourne participated in the study, and a subset of 29 refugees was interviewed after the one-year period. The phone number provided had many call categories, such as *Translating and Interpreting Service (TIS)*, *participants from the same community,* and *the training facilitator researchers* (Koh et al., 2018). Because many refugees struggle with the host language, groups were divided to share a same-language proficiency for oral communication and by culture of origin. The first six weeks, weekly training sessions were conducted, and the consequent five weeks, five bi-monthly training sessions were conducted. The goal of the training sessions was to improve communication skills with community interpreters.

The study utilized mixed methods and interviewed a subset of the refugees regarding their perception of the intervention. Intra-community calls were calls made to people who belong to the same community, extra-community calls were calls made to people who do not belong to the same community but live in Australia, and overseas calls were calls made to people in other countries. Analysis of how the phone was used portrayed that intra-community calls represented social capital bonding. Indeed, in each community, the category of the intra-community had a higher number of calls and call durations, compared to the other call categories. The thematic analysis of the interviews found that the perceived effects of the phone were social capital on an extra-community level; the intervention was shown to be beneficial in increasing refugees’ interactions with the Australian wider society. According to the refugees, skills taught in the intervention, such as confidence and communications skills, facilitated their interactions with the general host country society (Koh et al., 2018).

An interesting effect of the intervention was the strengthening of the community relationships of the mothers. Refugee mothers helped each other through childcare services and driving each other when needed. Furthermore, those with better English language skills would help the ones struggling with the language. The phone improved their social network, which became a source for emotional support and information access. The training sessions happened with community interpreters, and groups were divided as a function of shared mother tongue. This is beneficial as language is an important barrier for refugees (Green, 2017; Watkins et al., 2012). The study being conducted in the refugees' mother tongue, and community interpreters being present increases the validity of the findings. Secondly, the intervention focused on community building, which seems to be a protective factor in the interventions reviewed thus far. Finally, the intervention created a network where the refugees helped and supported each other and were each other’s language liaison. The creation of a bond of trust when it comes to information access is important. For example, Persian and Syrian refugees in Germany trust information provided by people in their own social network, who have successfully resettled, the most (Borkert et al., 2018).

## Discussion

Refugees are at a highly elevated risk of developing mental illness due to the adverse experiences and instability brought about by the resettlement process (Agic et al., 2016; Crowley, 2009). Moreover, refugee women, and especially refugee mothers, find themselves at a greater risk than their male counterparts to develop psychiatric symptoms and mental health problems (e.g. Rahman & Hafeez, 2003; Schweitzer et al., 2018; United Nations High Commissioner for Refugees et al., 2016). Thus, the current review sought to evaluate existing interventions aimed at supporting the specific needs and well-being of refugee mothers. Despite considerable evidence in the literature demonstrating the need for such interventions, only a limited number of interventions were found.

Many interventions involving refugee mothers focus on children and youth or on the family unit as a whole, seldom assess and address the mothers’ needs appropriately, and include little consideration for the mother’s psychological needs and well-being.

The present review suggests that across different populations and methodologies a pattern emerged whereby contact with others with similar experience and culture was essential (Koh et al., 2018; O’Shaughnessy et al., 2012; White et al., 2009). The Moving Forward Project, Sweet Mother and The Use of Mobile Phone for Social Capital Development interventions portray that community building and safe community spaces are protective factors for refugee mothers (Koh et al., 2018; O’Shaughnessy et al., 2012; White et al., 2009). The Moving Forward Project sessions provided the mothers with a safe space and what was described as a *gathering* (White et al., 2009), to connect with fellow refugees and simply talk, laugh, and bond. Similarly, the mothers experienced a new sense of *togetherness* in The Sweet Mothers intervention (O’Shaughnessy et al., 2012). Furthermore, refugee mothers felt that The Use of Mobile Phone for Social Capital Development intervention enhanced their relationship with their fellow community members, which provided them a space for emotional support and information access, and a network for exchanged childcare help, and consequently made their life “easier and better” (Koh et al., 2018). This is consistent with previous literature which has demonstrated that connection with those with similar experiences, values, and cultures is an essential aspect of social support (Chester, 1992; Kim et al., 2008; Stewart et al., 2008), which in turn is known to be a protective factor when struggling with mental health issues (e.g., Hefner & Eisenberg, 2009; Kawachi & Berkman, 2001; Watkins & Hill, 2018). Social support from refugees of similar backgrounds and experiences is a critical part of positive integration for refugee women, as it helps them build social capital in the post-resettlement country, allows them to socialize and express themselves, and thus relieve stress. Moreover, group learning in interventions focusing on social support and community building provides refugee women with otherwise scarce opportunities to build new relationships and better integrate in the host society (Saksena & McMorrow, 2020).

Furthermore, the refugee mothers benefited from having a safe space to discuss their shared experiences and challenges (O’Shaughnessy et al., 2012; Paris & Bronson, 2006). Having the chance to speak on the phone with members of their own community, in The Use of Mobile Phone for Social Capital Development intervention, or meeting with their community weekly, in the Moving Forward Project, was a positive experience for them (Koh et al., 2018; White et al., 2009). This safe space is especially valuable for refugees as they often struggle to discuss their difficulties in the host country because of various challenges including language barriers, complex intergroup relations, and the lack of access to a platform (Hansen & Houston, 2016). Moreover, creating safe spaces where refugee women can communicate while being physically and emotionally safe is central in helping them build a social network, receive social support, and learn important skills and information from women with similar experiences (United Nations High Commissioner for Refugees, 2014c). Similarly, the United Nation’s International Organization for Migration (IOM) has organized safe spaces for women and girls to come together, express themselves safely and openly, and develop positive coping strategies (International Organization for Migration, 2018). The use of such women-centered safe spaces for refugee women and girls has been shown to have positive impacts on participants’ mental health and well-being (Jahan Seema & Rahman, 2020). Thus, creating safe spaces for refugee women and girls is an effective way of supporting their post-resettlement needs and improving their mental health, well-being, and resilience through community-building.

The review also suggests that having allies increased refugees’ willingness to connect more to the host country society (Koh et al., 2018; O’Shaughnessy et al., 2012; Paris & Bronson, 2006). Mothers expressed feeling safer to honestly discuss their situation and experiences relating to resettlement within the context of the Sweet Mother intervention than with other professionals in different settings (O’Shaughnessy et al., 2012). This is worthwhile because newly resettled mothers do not tend to seek professional services because of barriers such as stigma, and lack of knowledge of community services and resources (Nicholson et al., 1998). In addition, having an ally to advocate for them, such as the Visiting Moms intervention working alliance, or the strengthened relationships that were formed because of The Use of Mobile Phone for Social Capital Development intervention helped improve the refugee mothers’ daily life and increased their interactions with the extra-community or host country wider society (Koh et al., 2018; Paris & Bronson, 2006). Indeed, community-building in the post-resettlement context leads to better access to health services, and increased access to adequate professional response in cases of gender-based violence (Jahan Seema & Rahman, 2020). Moreover, community programs and safe spaces can be used as an entry point by healthcare professionals to reach refugee women, mothers, and girls, build trust and rapport with the community, educate them on available services and health literacy, and better understand their needs (Abbasova, 2017; Shrivastava et al., 2017).

### Limitations and Future Directions

The review only includes scientific articles, published articles, and articles written in English. Plus, the review includes very few studies with great variability which makes generalization across studies difficult. In the future, there needs to be a careful consideration of the creation of safe spaces, advocacy, and community building in the conception of future interventions that aim to improve the well-being of refugee mothers. Future studies evaluating interventions for refugee mothers need to also be evaluated in more systematic ways.

### Conclusions and Practical Implications

While refugee mothers are at risk because of various factors, there are not many interventions that aim to meet their specific needs and improve their prognosis and well-being. Even though there are some interventions that incorporate refugee mothers’ needs and have positive impacts, they either do not calculate and assess outcomes, or the interventions’ methodology does not properly align with their outlined objectives. There is a concern for validity in interventions with refugees that conduct face-to-face training or interviewing in English when refugees have language barriers.

This systematic review suggests that emergent protective factors for refugee mothers are creating and providing a safe space, being a linguistic liaison, advocating for refugee mothers’ needs, and community-building. Thus, it would be helpful if practitioners and professionals working with refugee mothers educated them and informed them of accessible resources through pamphlets written in their native language, especially those that could enhance community-building and provide safe spaces. Indeed, providing refugee mothers with resources that can connect them to refugees from their own community or culture, rather than general refugee populations, is more helpful for them as they can use their own language or practice their own culture. Furthermore, whatever the institution, having professionals and front-line workers who can speak refugees’ native language seems to be imperative for the refugees’ knowledge of their rights, the resources they have access to, and the ways in which they can start connecting with the host country’s general community.
